# Accelerating turbo similarity searching in chemoinformatics on multicore and GPU platforms

**DOI:** 10.1186/1758-2946-6-S1-P38

**Published:** 2014-03-11

**Authors:** Marwah Haitham Al Laila, Nurul Malim, Nur’Aini Abdul Rashid

**Affiliations:** 1 School of Computer Sciences, Universiti Sains Malaysia, 11800, Penang, Malaysia

## 

The increase in the database size of chemical compound requires a longer time in processing for any searching algorithms [1]. With the focus on an algorithm called Turbo Similarity Searching which have been proven to have good accuracy in retrieving actives [2], we propose that this algorithm be implemented on the widely-used many-cores and multi-cores processors which are easily available at lower cost. This would help medicinal chemist runs virtual screening faster while maintaining the accuracy.

The many-cores processors are on-chip processors that could run simultaneously at a clock-cycle. Whilst the multi-cores processors are originally being developed to support graphics processing hence being called Graphics Processing Unit (GPU). However, the usage of GPU is now being generalized to include other general purpose operation [3]. Many works on the parallel field have tried implementing computational algorithms on this unit [4].

Taken into consideration on the compute intensive characteristics of TSS, we investigate the best method to parallelize it for better execution time. TSS is a two-phase algorithm in which its second phase is a compute intensive portion of the algorithm. Hence, if this phase could be accelerated, TSS would run faster and searching through a larger database would not be less a problem. This poster describes our experimental details and results on the matter.

## References

[B1] Pu Liu DKA Yang E Accelerating Chemical Database Searching Using Graphics Processing Units J Chem. Inf. Model 2011 51 1807 1816 10.1021/ci200164g 21696144

[B2] Malim N Enhancing Similarity Searching PhD Thesis 2011 University of Sheffield, UK

[B3] Harish P Narayanan P Accelerating large graph algorithms on the GPU using CUDA High Performance Computing–HiPC 2007 197 208

[B4] Maggioni M Santambrogio MD Liang J GPU-accelerated Chemical Similarity Assessment for Large Scale Databases Procedia Computer Science 2007 4 2007 2016 10.1016/j.procs.2011.04.219 PMC5072535 27774113

